# Association Between Repeated Exposure to Hurricanes and Mental Health in a Representative Sample of Florida Residents

**DOI:** 10.1001/jamanetworkopen.2022.17251

**Published:** 2022-06-16

**Authors:** Dana Rose Garfin, Rebecca R. Thompson, E. Alison Holman, Gabrielle Wong-Parodi, Roxane Cohen Silver

**Affiliations:** 1 Sue & Bill Gross School of Nursing, University of California, Irvine; 2 Program in Public Health, University of California, Irvine; 3 Department of Psychological Science, University of California, Irvine; 4 Earth System Science and Stanford Woods Institute for the Environment, Stanford University, Stanford, California; 5 Department of Medicine, University of California, Irvine

## Abstract

**Importance:**

During the past century, more than 100 catastrophic hurricanes have impacted the Florida coast; climate change will likely be associated with increases in the intensity of future storms. Despite these annual threats to residents, to our knowledge, no longitudinal studies of representative samples at risk of hurricane exposure have examined psychological outcomes associated with repeated exposure.

**Objective:**

To assess psychosocial and mental health outcomes and functional impairment associated with repeated hurricane exposure.

**Design, Setting, and Participants:**

In this survey study, a demographically representative sample of Florida residents was assessed in the 60 hours prior to Hurricane Irma (wave 1: September 8-11, 2017). A second survey was administered 1 month after Hurricane Irma (wave 2: October 12-29, 2017), and a third survey was administered after Hurricane Michael (wave 3: October 22 to November 6, 2018). Data were analyzed from July 19 to 23, 2021.

**Exposure:**

Hurricanes Irma and Michael.

**Main Outcomes and Measures:**

The main outcomes were posttraumatic stress symptoms (PTSS), global distress, worry about future events (generalized worries), and functional impairment. Path models were used to assess associations of individual-level factors (prior mental health, recent adversity), prior storm exposures (loss and/or injury, evacuation), and direct, indirect, and media-based exposures to hurricanes Irma and Michael with those outcomes. Poststratification weights were applied to facilitate population-based inferences.

**Results:**

Of 2873 individuals administered the survey in wave 1, 1637 responded (57.0% completion rate) (894 [54.6%, weighted] women; mean [SD] age, 51.31 [17.50] years); 1478 in wave 2 (90.3% retention from wave 1) and 1113 in wave 3 (75.3% retention from wave 2) responded. Prior mental health ailments (b, 0.18; 95% CI, 0.07-0.28), prior hurricane-related loss and/or injury (b, 0.09; 95% CI, 0.02-0.17), hours of Hurricane Irma–related media exposure (b, 0.03; 95% CI, 0.02-0.04), being in an evacuation zone during Hurricane Irma and not evacuating (b, 0.14; 95% CI, 0.02-0.27), and loss and/or injury in Hurricane Irma (b, 0.35; 95% CI, 0.25-0.44) were positively associated with PTSS after Hurricane Irma; most associations persisted and were associated with responses to Hurricane Michael. Prior mental health ailments (b, 0.10; 95% CI, 0.03-0.17), hours of Hurricane Michael–related media exposure (b, 0.01; 95% CI, 0.003-0.02), hurricane Irma-related PTSS (b, 0.42; 95% CI, 0.34-0.50), recent individual-level adversity (b, 0.03; 95% CI, 0.005-0.05), being in an evacuation zone during Hurricane Irma and evacuating (b, 0.10; 95% CI, 0.002-0.19), and direct (b, 0.36; 95% CI, 0.16-0.55) and indirect (b, 0.12; 95% CI, 0.05-0.18) Hurricane Michael–related exposures were directly associated with Hurricane Michael–related PTSS. After Hurricane Michael, prior mental health ailments (b, 0.17; 95% CI, 0.06-0.28), and PTSS related to hurricanes Irma (b, 0.11; 95% CI, 0.001-0.22) and Michael (b, 0.58; 95% CI, 0.47-0.69) were associated with respondents’ functional impairment. Analogous analyses using global distress and generalized worries as mediators of functional impairment yielded a similar pattern of results.

**Conclusions and Relevance:**

In this survey study, repeated direct, indirect, and media-based exposures to hurricanes were associated with increased mental health symptoms among Florida residents who experienced hurricanes Irma and Michael, suggesting that people were sensitized to respond with more psychological symptoms over time. These results may inform targeted public health intervention efforts for natural disasters.

## Key Points

**Question:**

What psychological outcomes are associated with repeated exposure to catastrophic hurricanes?

**Findings:**

In this survey study of 1637 Florida residents, repeated direct, indirect, and media exposures to hurricanes Irma and Michael were positively associated with posttraumatic stress symptoms, generalized worries, global distress, and functional impairment. Individual-level factors (prior mental health ailments), storm exposure factors (loss and/or injury, evacuation), knowing someone directly exposed, and media exposure to the hurricanes were associated with ongoing symptoms.

**Meaning:**

The findings suggest that repeated exposure to hurricanes sensitizes people to respond with more psychological symptoms over time and may be associated with increased mental health risks.

## Teaser

This survey study examines the association between repeated hurricane exposure and mental health and functional impairment outcomes among Florida residents exposed to hurricanes Irma and Michael.

## Introduction

Hurricanes, like many other natural hazards, threaten specific communities annually. In 2017, when Hurricane Irma approached Florida as a category 5 storm, 6.5 million people were put under mandatory evacuation orders.^1^ Images of a giant superstorm threatening the densely populated coast dominated the media. Damages cost more than $50 billion, making it one of the most expensive storms in US history.^1,2,3^ One year later, Hurricane Michael (category 5), one of the strongest hurricanes in Florida’s history, made landfall on the Florida panhandle with 160-mph winds and a 9- to 14-ft storm surge, resulting in 16 deaths and $25 billion in damages.^3,4^ Widespread media coverage broadcast the threat of both storms statewide because of the evolving risk of direct exposure.^5^ These storms are not exceptions; more than 100 catastrophic hurricanes made landfall in Florida in the past century. Climate change will likely increase the intensity of subsequent storms.^4,6^

Hurricane exposure correlates with psychological distress,^7^ and storm severity correlates with posttraumatic stress disorder.^8^ After Hurricane Katrina, psychopathologic symptoms increased, highlighting the potential long-term associations between such exposures and mental health.^7^ Despite the annual hurricane season threat, to our knowledge, no longitudinal studies have examined psychological responses associated with repeated hurricane exposure, and media exposure has rarely been incorporated. Few studies^9^ included prestorm assessments of psychological symptoms. Even methodologically rigorous studies often used cross-sectional designs or retrospective reports of prestorm experiences.^10^ Thus, little is known about how factors assessed before hurricanes, acute psychological responses, and repeated hurricane exposure may be associated with longitudinal mental health outcomes.

Despite methodological limitations, prior research provides insight into key factors associated with psychological responses. Factors including prior mental health ailments^11^ and demographic indicators (ie, educational level, socioeconomic status) were correlated with poor self-rated mental health after hurricanes Harvey^12^ and Katrina^13^ (although other researchers reported divergent Hurricane Katrina findings^14^). Predisaster traumas^15^ that elicited adverse responses^16^ and prior disaster exposures^15,17,18^ also correlated with postevent mental health. Direct storm-related exposure variables (eg, displacement, financial loss, and property damage) have been associated with adverse psychological responses, particularly posttraumatic stress symptoms (PTSS).^19^ Controlling for direct storm exposure, analyses found that media-based hurricane exposure was associated with distress^9,20,21^ and may have additive effects.^22^ Importantly, while habituation effects of adversity are plausible,^23^ robust research suggests that cumulative adversity exposure (including cascading collective traumas^24^ and disaster-related secondary stressors^25^) is associated with worse outcomes over time.

This study used a rare design with epidemiological assessments collected immediately before an approaching storm (Hurricane Irma) and immediately after 2 major Florida hurricanes (Irma and Michael) that occurred in annual succession; mental health ailments assessed before Hurricane Irma were also prospectively collected. Using a population-based representative sample of Florida adults surveyed 3 times during a 2-year period when 2 devastating hurricanes made landfall in Florida, we explored factors associated with mental health and functional impairment. We hypothesized that (1) individual-level factors (demographics, prestorm mental health, and recent adversity), storm-related exposures (evacuation status, property loss, and direct or indirect injury), and media exposure would be associated with worse short- and longer-term psychological outcomes and (2) short-term responses would be associated with longer-term psychological outcomes that would correlate with functional impairment after a subsequent hurricane.

## Methods

In this survey study, participants were from the GfK (now Ipsos) KnowledgePanel, which was designed to be representative of US residents. Ipsos uses address-based sampling to randomly recruit panelists using probability-based sampling methods, and it collects and updates KnowledgePanel participants’ information regularly. Households without an internet connection are provided internet access. Ipsos emails panelists the links to surveys, which are completed on computers or mobile devices. This study’s sampling frame was Florida residents. The institutional review board of the University of California, Irvine approved all procedures; respondents were considered to have provided informed consent by completing the surveys after reading a brief introduction describing the study. Participants received $15 to $20 compensation for completing each 15- to 20-minute survey. The study followed the American Association for Public Opinion Research (AAPOR) reporting guideline^26,27^ and the Strengthening the Reporting of Observational Studies in Epidemiology (STROBE) reporting guideline.^28^

The wave 1 survey was fielded to all Florida panelists before Hurricane Irma’s landfall, between 6 pm on September 8, 2017, and 3 pm on September 11, 2017. One month after Irma (October 12-29, 2017), respondents to the wave 1 survey were administered a second survey (wave 2). An earlier report presents results from the first 2 waves of this longitudinal study.^9^ In the current study, approximately 1 year after the wave 2 survey, a third survey (wave 3) was fielded to the sample 2 to 3 weeks after Hurricane Michael (October 22 to November 6, 2018), a category 5 storm. The study was well powered (β > .80) to detect small effects (*f*^2^ = 0.02) for 22 variables.

### Measures

#### Individual-Level Characteristics

Prior to the wave 1 survey, Ipsos collected responses to an item from the National Center for Health Statistics annual National Health Interview Survey^29^: “Has a medical doctor ever diagnosed you as suffering from any of the following ailments?” Prompts were depression and anxiety disorders. Comparisons between responses to the National Health Interview Survey item and the KnowledgePanel survey supported data validity (<1.5% difference).^30^ Missing values (4.5% of the sample) were imputed using sequential hot deck imputation.^31,32^

In the wave 3 survey, participants reported past-year experience with 37 adverse events (eg, serious accident or injury, domestic violence).^33^ Items were coded as 0 (“did not occur”) or 1 (“occurred”) and summed.

#### Hurricane-Related Exposures

In wave 1, previous hurricane-related evacuation zone experience included (1) evacuated, (2) did not evacuate, or (3) wanted to evacuate but could not. Responses were dichotomized as 0 (“no experience”) or 1 (“at least 1 experience”). Prior direct (eg, lost home or property, injured, or lost a pet) and indirect (eg, knowing someone injured or killed) hurricane exposures were summed.

In waves 1 and 3, daily hours (0 to ≥11) spent engaged with hurricane-related (1) television, radio, or print; (2) online news sources (CNN, NYTimes.com); and (3) social media (eg, Facebook, Twitter) in the days since coverage began were summed. In wave 2, evacuation experience during Hurricane Irma was coded as 0 (“not in an evacuation zone”), 1 (“evacuated”), or 2 (“in an evacuation zone but did not evacuate”). The number of direct (eg, lost home or property, injured, and lost a pet) and indirect (knowing someone injured or killed) exposures during Hurricane Irma were summed. Direct exposure to Hurricane Michael was assessed at wave 3; the number of losses (eg, lost home or property, injured, or lost a pet) and evacuation status during Hurricane Michael were summed.

Indirect exposure to Hurricane Michael was assessed by asking respondents to report if they knew someone who experienced a loss, was near the path of the storm, or was injured or killed during Hurricane Michael. Because direct exposure to Hurricane Michael was geographically limited to the Florida panhandle, indirect exposure to Hurricane Michael was assessed more comprehensively. Direct and indirect exposures to Hurricane Michael were treated as separate variables.

### Outcome Variables

For waves 2 and 3, we used a modified version of the Primary Care Posttraumatic Stress Disorder Screen for the *Diagnostic and Statistical Manual of Mental Disorders* (Fifth Edition)^34,35^ that was implemented in prior research^9,33,36^ to assess prior-week hurricane-related PTSS using a 5-point scale (1 [“never”] to 5 [“all the time”]; wave 2: α = .87; wave 3: α = .83). These modifications capture variability in an inherently dimensional construct^37^ assessed in respondents exposed directly and indirectly via a close other person (criterion A) and through the media (not criterion A). Fielding our survey soon after the hurricanes also required a shorter time frame (ie, past week) to avoid overlap with possible prehurricane symptoms.

In waves 2 and 3, a 9-item version of the Brief Symptom Inventory–18^38^ was used to measure global distress. Respondents reported anxiety, depression, and somatization symptoms in the prior 7 days (0 [“not at all”] to 4 [“extremely”]; wave 2: α = .90; wave 3: α = .89).

Eight items from previous research^39^ were used to assess ongoing past-week worries about the possibility of (1) terrorist attacks, (2) natural disasters, (3) violence (shootings, stabbings, or physical assault), and (4) financial stress or strain (1 [“never”] to 5 [“all of the time”]; wave 2: α = .90; wave 3: α = .90). In wave 3, 4 items from the 36-Item Short-Form Health Survey^40^ were used to assess physical and emotional health (1 [“none of the time”] to 5 [“all the time”]; α = .89).

### Statistical Analysis

**Figure 1.**
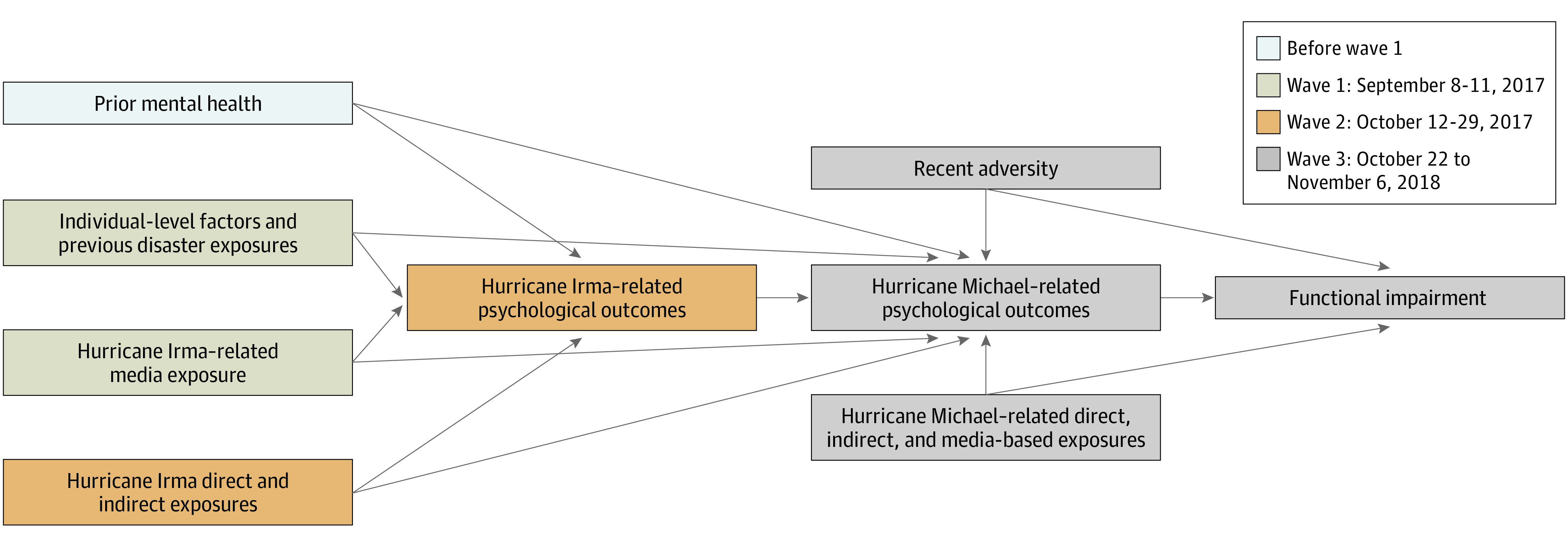
Theoretical Model of Hypothesized Associations Between Prestorm Factors, Storm Exposures, Posthurricane Psychological Outcomes, and Functional Impairment

Data were analyzed from July 19 to 23, 2021. Using Stata, version 16.1 (StataCorp LLC), 3 path models (1 for each dependent variable) tested the associations of individual-level factors (eg, demographics, prior mental health, and recent adversity); prior storm exposures (evacuation, storm-related loss and/or injury); and direct (personal evacuation, storm-related loss and/or injury), indirect (storm-related loss and/or injury of a close other person), and media-based exposures to hurricanes Irma and Michael with PTSS (waves 2 and 3), ongoing generalized worries (waves 2 and 3), and global distress (waves 2 and 3). Functional impairment in wave 3 was the final outcome. Figure 1 shows the hypothesized model. Significance was measured as 2-sided *P* < .05. Poststratification weights accounted for differential probabilities of panel recruitment and adjusted the final sample to US census benchmarks for Florida. Weights were constructed iteratively from panel-level design weights and included gender, age, race and ethnicity, household income, residing in a metropolitan or nonmetropolitan area, and educational level.

For missing data across and within waves, Little’s Missing Completely at Random Test was implemented.^41^ Results suggested that data were missing completely at random (χ^2^_522_ = 554.83; *P* = .16). Thus, full information maximum likelihood was implemented using all available data within and between waves.^42^ Robust SEs are presented as appropriate for complex survey data. For individual scales, because of the low rate of missing data (<5% on any item), row mean substitution (by subscale, if applicable) was implemented if respondents answered more than 50% of questions per measure. This produces the least amount of bias compared with other approaches^43^ and is consistent with analyses of similar data sets.^22,44,45^

## Results

Of 2873 Florida residents administered the wave 1 survey, 1637 responded (57.0% AAPOR-defined completion rate^26,27^), 1579 (96.5%) of whom responded within 48 hours; 1478 respondents completed the wave 2 survey (90.3% retention from wave 1). Ninety-five participants who did not complete wave 1 but had participated in another ongoing study were invited to participate in wave 2; 40 of these completed the survey, for a total of 1518 wave 2 participants. These 40 were not included in the inferential statistics or in the completion or retention rates. In wave 3, 1113 people completed the survey (75.3% retention from wave 2; 66.7% retention from wave 1).

Among the 1637 respondents in the total sample, the mean (SD) age was 51.31 (17.50) years and 894 (54.6%, weighted) were women. The wave 1 weighted sample demographics were close to US census benchmarks for Florida (eTable 1 in the Supplement). A total of 1369 participants (83.6%) reported no prior mental health diagnoses, 186 (11.4%) reported a previous depression or anxiety diagnosis, and 81 (5.0%) reported both diagnoses.

A map of participants’ locations across the state of Florida is presented in eFigure 1 in the Supplement. All numbers and percentages are weighted and may vary slightly owing to missing data and rounding. Of 1637 wave 1 participants, 389 (24.5%) had at least 1 evacuation experience before Hurricane Irma; 300 (18.4%) experienced a hurricane-related loss before Hurricane Irma. The mean (SD) amount of Hurricane Irma–related media exposure was 7.91 (7.33) hours across all sources (3.84 [3.30] hours of television, radio, and print news; 2.19 [2.84] hours of online news; and 1.93 [2.90] hours of social media). Of 1518 wave 2 participants, 756 (50.0%) reported being in an evacuation zone during Hurricane Irma, and 193 (12.7%) experienced a Hurricane Irma–related loss and/or injury. Of 1113 wave 3 participants, 117 (10.5%) reported direct Hurricane Michael exposure, 101 (9.1%) reported being in an evacuation zone, and 406 (36.6%) reported indirect exposure to Hurricane Michael. The mean (SD) amount of Hurricane Michael–related media exposure was 4.92 (5.85) hours across all sources (2.37 [2.76] hours of television, radio, and print news; 1.38 [2.13] hours of online news; and 1.18 [2.28] hours of social media). A total of 1582 respondents (96.7%) lived in a metropolitan area,^46^ consistent with the state population. Descriptive statistics for the dependent constructs are presented in eTable 2 in the Supplement.

**Figure 2.**
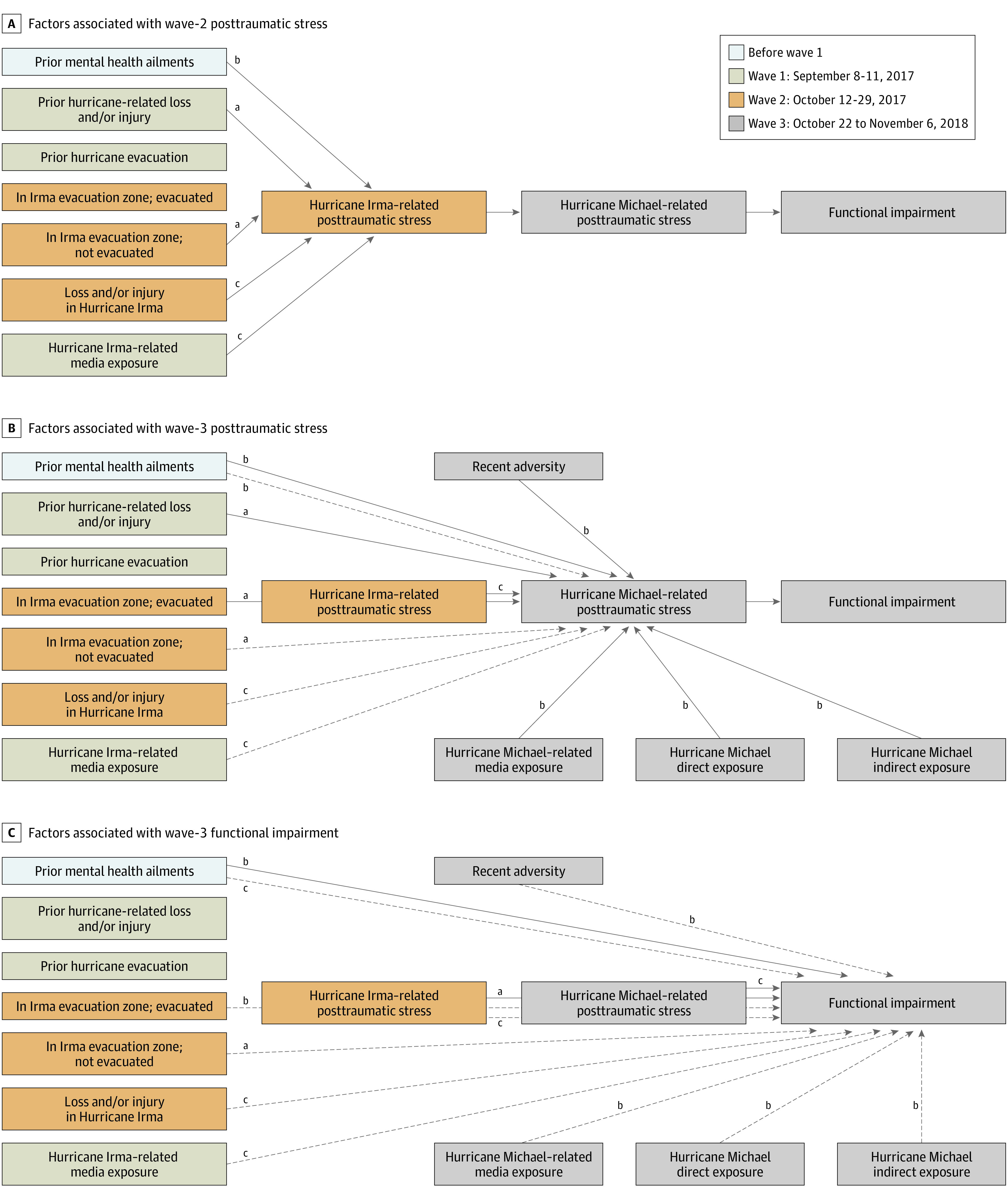
Direct and Indirect Associations Between Prestorm Factors, Storm Exposures, Posthurricane Posttraumatic Stress, and Functional Impairment Solid lines represent direct associations and dashed lines, indirect associations. ^a^*P* < .05. ^b^*P* < .01. ^c^*P* < .001.

Table 1 presents factors associated with hurricane-related PTSS over time. Pre–Hurricane Irma mental health ailments (b, 0.18; 95% CI, 0.07-0.28), prior hurricane-related loss and/or injury (b, 0.09; 95% CI, 0.02-0.17), Hurricane Irma–related media exposure (b, 0.03; 95% CI, 0.02-0.04), being in an evacuation zone and not evacuating during Hurricane Irma (b, 0.14; 95% CI, 0.02-0.27), and Hurricane Irma–related loss and/or injury (b, 0.35; 95% CI, 0.25-0.44) were directly associated with a linear increase in PTSS after Hurricane Irma (Figure 2A). The following were directly associated with increased PTSS after Hurricane Michael: mental health ailments before Hurricane Irma (b, 0.10; 95% CI, 0.03-0.17), being in an evacuation zone and evacuating during Hurricane Irma (b, 0.10; 95% CI, 0.002-0.19), hours of Hurricane Michael–related media exposure (b, 0.01; 95% CI, 0.003-0.02), both direct (b, 0.36; 95% CI, 0.16-0.55) and indirect (b, 0.12; 95% CI, 0.05-0.18) exposure to Hurricane Michael, recent individual-level adversity (b, 0.03; 95% CI, 0.005-0.05), and wave 2 PTSS (b, 0.42; 95% CI, 0.34-0.50). Hurricane-related loss and/or injury before Hurricane Irma, pre-Hurricane Irma mental health ailments, loss and/or injury in Hurricane Irma, Hurricane Irma–related media exposure, and being in an evacuation zone but not evacuating during Hurricane Irma were indirectly associated with higher number of Hurricane Michael–related PTSS through a higher number of early PTSS after Hurricane Irma (Figure 2B). Pre–Hurricane Irma mental health ailments and wave 2 and 3 PTSS were directly associated with higher symptoms of functional impairment. Increased hours of Hurricane Irma–related media, pre-Hurricane Irma mental health ailments, being in an evacuation zone and evacuating or not evacuating during Hurricane Irma, Hurricane Irma–related loss and/or injury, hours of Hurricane Michael–related media exposure, direct and indirect exposure to Hurricane Michael, recent individual-level adversity, and wave 2 PTSS were indirectly associated with higher symptoms of functional impairment (Figure 2C).

**Table 1.** Path Model of Factors Associated With PTSS After Hurricanes Irma and Michael and Functional Impairment 1 Year After Hurricane Michael^a^

Variable	b (95% CI)					
	Wave 2 (after Hurricane Irma): PTSS		Wave 3 (after Hurricane Michael)			
			PTSS		Functional impairment	
	Direct	Indirect	Direct	Indirect	Direct	Indirect
**Wave 1**						
Mental health ailments before Hurricane Irma^b^	0.18 (0.07 to 0.28)^c^	NA	0.10 (0.03 to 0.17)^c^	0.07 (0.03 to 0.12)^c^	0.17 (0.06 to 0.28)^c^	0.12 (0.05 to 0.19)^d^
Loss and/or injury before Hurricane Irma	0.09 (0.02 to 0.17)^e^	NA	0.02 (−0.05 to 0.09)	0.04 (0.01 to 0.07)^e^	−0.04 (−0.12 to 0.05)	0.05 (−0.002 to 0.10)
Evacuation experience before Hurricane Irma	0.06 (−0.09 to 0.20)	NA	0.03 (−0.07 to 0.12)	0.02 (−0.04 to 0.08)	−0.06 (−0.18 to 0.06)	0.03 (−0.04 to 0.11)
Hours of Hurricane Irma–related media exposure	0.03 (0.02 to 0.04)^d^	NA	0.01 (−0.003 to 0.02)	0.01 (0.01 to 0.02)^d^	0.0003 (−0.01 to 0.01)	0.01 (0.01 to 0.02)^d^
Female^f^	0.07 (−0.03 to 0.17)	NA	−0.01 (−0.09 to 0.08)	0.03 (−0.01 to 0.07)	0.08 (−0.01 to 0.18)	0.02 (−0.03 to 0.08)
College education	−0.002 (−0.09 to 0.08)	NA	0.01 (−0.07 to 0.09)	−0.001 (−0.04 to 0.03)	−0.05 (−0.15 to 0.04)	0.01 (−0.05 to 0.06)
Race and ethnicity^g^						
Black, non-Hispanic	−0.10 (−0.23 to 0.03)	NA	0.16 (−0.02 to 0.34)	−0.04 (−0.10 to 0.01)	−0.03 (−0.22 to 0.16)	0.06 (−0.06 to 0.18)
Hispanic	0.09 (−0.04 to 0.22)	NA	0.002 (−0.09 to 0.10)	0.04 (−0.02 to 0.09)	−0.05 (−0.16 to 0.06)	0.03 (−0.04 to 0.10)
Other, non-Hispanic^h^	0.19 (−0.04 to 0.42)	NA	0.03 (−0.16 to 0.21)	0.08 (−0.02 to 0.18)	−0.20 (−0.44 to 0.03)	0.08 (−0.08 to 0.24)
Income	−0.03 (−0.04 to −0.02)^d^	NA	−0.01 (−0.02 to 0.01)	−0.01 (−0.02 to −0.01)^d^	−0.01 (−0.02 to 0.001)	−0.01 (−0.02 to −0.005)^c^
**Wave 2**						
In evacuation zone during Hurricane Irma and did not evacuate^i^	0.14 (0.02 to 0.27)^e^	NA	0.06 (−0.04 to 0.16)	0.06 (0.01 to 0.11)^e^	0.13 (−0.01 to 0.27)	0.08 (0.01 to 0.16)^e^
In evacuation zone during Hurricane Irma and evacuated^i^	0.10 (−0.02 to 0.22)	NA	0.10 (0.002 to 0.19)^e^	0.04 (−0.01 to 0.09)	0.02 (−0.08 to 0.13)	0.09 (0.02 to 0.16)^c^
Loss and/or injury in Hurricane Irma	0.35 (0.25 to 0.44)^d^	NA	−0.04 (−0.14 to 0.05)	0.14 (0.10 to 0.19)^d^	−0.02 (−0.12 to 0.07)	0.10 (0.03 to 0.16)^c^
Hurricane Irma–related PTSS	NA	NA	0.42 (0.34 to 0.50)^d^	NA	0.11 (0.001 to 0.22)^e^	0.24 (0.18 to 0.30)^d^
**Wave 3**						
Direct exposure to Hurricane Michael	NA	NA	0.36 (0.16 to 0.55)^d^	NA	0.001 (−0.21 to 0.21)	0.21 (0.09 to 0.32)^c^
Indirect exposure to Hurricane Michael	NA	NA	0.12 (0.05 to 0.18)^d^	NA	0.05 (−0.02 to 0.11)	0.07 (0.03 to 0.11)^c^
Hours of Hurricane Michael–related media exposure	NA	NA	0.01 (0.003 to 0.02)^c^	NA	0.01 (−0.002 to 0.02)	0.01 (0.002 to 0.01)^c^
Recent individual-level adversity	NA	NA	0.03 (0.005 to 0.05)^e^	NA	0.01 (−0.01 to 0.03)	0.02 (0.002 to 0.03)^e^
Hurricane Michael–related PTSS	NA	NA	NA	NA	0.58 (0.47 to 0.69)^d^	NA

Abbreviations: NA, not applicable; PTSS, posttraumatic stress symptoms.

^a^ Data are for 1637 Florida residents in the total sample. Florida residents were surveyed in the 60 hours before Hurricane Irma (wave 1: September 8-11, 2017). A second survey was administered 1 month after Hurricane Irma (wave 2: October 12-29, 2017), and a third survey was administered after Hurricane Michael (wave 3: October 22 to November 6, 2018).

^b^ A response of 0 indicated no prior mental health ailments; 1, prior anxiety or depression; and 2, prior anxiety and depression.

^c^ *P* < .01.

^d^ *P* < .001.

^e^ *P* < .05.

^f^ Male was the reference group.

^g^ White, non-Hispanic was the reference group.

^h^ Other included those who identified as American Indian or Alaska Native, Asian, Native Hawaiian or other Pacific Islander, or “a different race.”

^i^ Not in an evacuation zone was the reference group.

Table 2 presents factors associated with higher symptoms of ongoing generalized worries after hurricanes Irma and Michael. Pre–Hurricane Irma mental health ailments and hurricane-related loss and/or injury, hours of Hurricane Irma-related media exposure, being in an evacuation zone and not evacuating during Hurricane Irma, and Hurricane Irma–related loss and/or injury were directly associated with post–Hurricane Irma worries (eFigure 2 in the Supplement). Being in an evacuation zone and evacuating during Hurricane Irma, hours of Hurricane Michael–related media exposure, indirect exposure to Hurricane Michael, recent adversity, and post–Hurricane Irma worries were directly associated with worries after Hurricane Michael. Pre–Hurricane Irma mental health ailments and hurricane-related loss and/or injury, hours of Hurricane Irma–related media exposure, being in an evacuation zone and not evacuating during Hurricane Irma, and Hurricane Irma–related loss and/or injury were indirectly positively associated with worries after Hurricane Michael (eFigure 2 in the Supplement). Pre–Hurricane Irma mental health ailments, being in an evacuation zone and not evacuating during Hurricane Irma, direct exposure to Hurricane Michael, and post–Hurricane Michael worries were directly associated with greater functional impairment; indirect associations were identified for pre–Hurricane Irma mental health ailments, hours of Hurricane Irma–related media exposure, being in an evacuation zone and evacuating during Hurricane Irma, post–Hurricane Irma worries, indirect exposure to Hurricane Michael, Hurricane Michael–related media exposure, and recent individual-level adversity (eFigure 2 in the Supplement).

**Table 2.** Path Model of Factors Associated With Generalized Worries After Hurricanes Irma and Michael and Functional Impairment After Hurricane Michael^a^

Variable	b (95% CI)					
	Wave 2 (after Hurricane Irma): generalized worries		Wave 3 (after Hurricane Michael)			
			Generalized worries		Functional impairment	
	Direct	Indirect	Direct	Indirect	Direct	Indirect
**Wave 1**						
Mental health ailments before Hurricane Irma^b^	0.15 (0.03 to 0.27)^c^	NA	0.07 (−0.03 to 0.18)	0.08 (0.01 to 0.14)^c^	0.22 (0.11 to 0.33)^d^	0.07 (0.02 to 0.12)^e^
Loss and/or injury before Hurricane Irma	0.17 (0.09 to 0.26)^d^	NA	−0.04 (−0.14 to 0.07)	0.09 (0.05 to 0.14)^d^	−0.01 (−0.10 to 0.09)	0.03 (−0.02 to 0.08)
Evacuation experience before Hurricane Irma	0.11 (−0.07 to 0.29)	NA	−0.00002 (−0.12 to 0.12)	0.06 (−0.04 to 0.15)	−0.07 (−0.19 to 0.04)	0.03 (−0.04 to 0.09)
Hours of Hurricane Irma–related media exposure	0.03 (0.02 to 0.04)^d^	NA	0.001 (−0.01 to 0.01)	0.02 (0.01 to 0.02)^d^	0.003 (−0.005 to 0.01)	0.01 (0.003 to 0.01)^e^
Female^f^	0.14 (0.02 to 0.26)^c^	NA	0.10 (0.01 to 0.18)^c^	0.08 (0.01 to 0.14)^c^	0.04 (−0.06 to 0.14)	0.07 (0.03 to 0.12)^e^
College education	−0.07 (−0.18 to 0.04)	NA	0.08 (−0.004 to 0.17)	−0.04 (−0.09 to 0.02)	−0.06 (−0.15 to 0.04)	0.02 (−0.03 to 0.07)
Race and ethnicity^g^						
Black, non-Hispanic	0.001 (−0.23 to 0.23)	NA	0.02 (−0.16 to 0.20)	0.0005 (−0.12 to 0.12)	0.02 (−0.15 to 0.20)	0.01 (−0.09 to 0.10)
Hispanic	0.06 (−0.11 to 0.22)	NA	0.02 (−0.11 to 0.14)	0.03 (−0.06 to 0.12)	−0.05 (−0.18 to 0.07)	0.02 (−0.04 to 0.08)
Other, non-Hispanic^h^	0.26 (−0.05 to 0.58)	NA	0.07 (−0.26 to 0.40)	0.14 (−0.03 to 0.31)	−0.25 (−0.51 to 0.01)	0.10 (−0.05 to 0.24)
Income	−0.02 (−0.04 to −0.005)^c^	NA	−0.01 (−0.02 to 0.001)	−0.01 (−0.02 to −0.002)^c^	−0.02 (−0.03 to −0.003)^c^	−0.01 (−0.02 to −0.003)^e^
**Wave 2**						
In evacuation zone during Hurricane Irma and did not evacuate^i^	0.16 (0.01 to 0.31)^c^	NA	0.02 (−0.11 to 0.15)	0.09 (0.002 to 0.17)^c^	0.17 (0.03 to 0.31)^c^	0.05 (−0.02 to 0.11)
In evacuation zone during Hurricane Irma and evacuated^i^	0.10 (−0.06 to 0.25)	NA	0.13 (0.02 to 0.24)^c^	0.05 (−0.03 to 0.13)	0.05 (−0.06 to 0.17)	0.08 (0.02 to 0.14)^e^
Loss and/or injury in Hurricane Irma	0.23 (0.12 to 0.34)^d^	NA	−0.03 (−0.14 to 0.08)	0.12 (0.06 to 0.18)^d^	0.03 (−0.08 to 0.14)	0.04 (−0.01 to 0.10)
Generalized worries	NA	NA	0.53 (0.46 to 0.61)^d^	NA	0.03 (−0.06 to 0.12)	0.22 (0.16 to 0.28)^d^
**Wave 3**						
Direct exposure to Hurricane Michael	NA	NA	0.11 (−0.08 to 0.30)	NA	0.21 (0.003 to 0.41)^c^	0.05 (−0.03 to 0.12)
Indirect exposure to Hurricane Michael	NA	NA	0.13 (0.07 to 0.19)^d^	NA	0.06 (−0.01 to 0.13)	0.05 (0.02 to 0.08)^e^
Hours of Hurricane Michael–related media exposure	NA	NA	0.01 (0.003 to 0.02)^e^	NA	0.01 (−0.0005 to 0.03)	0.01 (0.001 to 0.01)^c^
Recent individual-level adversity	NA	NA	0.03 (0.01 to 0.06)^e^	NA	0.02 (−0.004 to 0.04)	0.01 (0.004 to 0.02)^e^
Generalized worries	NA	NA	NA	NA	0.41 (0.31 to 0.51)^d^	NA

^a^ Data are for 1637 Florida residents in the total sample. Florida residents were surveyed in the 60 hours before Hurricane Irma (wave 1: September 8-11, 2017). A second survey was administered 1 month after Hurricane Irma (wave 2: October 12-29, 2017), and a third survey was administered after Hurricane Michael (wave 3: October 22 to November 6, 2018).

^b^ A response of 0 indicated no prior mental health ailments; 1, prior anxiety or depression; and 2, prior anxiety and depression.

^c^ *P* < .05.

^d^ *P* < .001.

^e^ *P* < .01.

^f^ Male was the reference group.

^g^ White, non-Hispanic was the reference group.

^h^ Other included those who identified as American Indian or Alaska Native, Asian, Native Hawaiian or other Pacific Islander, or “a different race.”

^i^ Not in an evacuation zone was the reference group.

Table 3 presents factors associated with global distress over time. Pre–Hurricane Irma mental health ailments, hours of Hurricane Irma–related media exposure, being in an evacuation zone and not evacuating during Hurricane Irma, and Hurricane Irma–related loss and/or injury were directly associated with higher symptoms of global distress in wave 2 (eFigure 3 in the Supplement). Recent adversity and global distress in wave 2 were significantly associated with higher symptoms of global distress in wave 3. Pre–Hurricane Irma mental health ailments, hours of Hurricane Irma–related media exposure, being in an evacuation zone and not evacuating during Hurricane Irma, and Hurricane Irma–related loss and/or injury were indirectly associated with higher symptoms of global distress in wave 3 (eFigure 3 in the Supplement). For functional impairment in wave 3, direct associations were identified for being in an evacuation zone and not evacuating during Hurricane Irma, hours of Hurricane Michael–related media exposure, indirect exposure to Hurricane Michael, and higher symptoms of global distress in wave 3. Indirect associations with functional impairment in wave 3 were also identified for pre–Hurricane Irma mental health ailments, hours of Hurricane Irma–related media exposure, being in an evacuation zone (both evacuating and not evacuating) during Hurricane Irma, Hurricane Irma–related loss and/or injury, higher symptoms of global distress in wave 2, and recent individual-level adversity (eFigure 3 in the Supplement).

**Table 3.** Path Model of Factors Associated With Global Distress After Hurricanes Irma and Michael and Functional Impairment After Hurricane Michael^a^

Variable	b (95% CI)					
	Wave 2 (after Hurricane Irma): global distress		Wave 3 (after Hurricane Michael)			
			Global distress		Functional impairment	
	Direct	Indirect	Direct	Indirect	Direct	Indirect
**Wave 1**						
Mental health ailments before Hurricane Irma^b^	0.31 (0.21 to 0.41)^c^	NA	0.08 (−0.01 to 0.17)	0.17 (0.10 to 0.23)^c^	0.09 (−0.01 to 0.20)	0.22 (0.11 to 0.33)^c^
Loss and/or injury before Hurricane Irma	0.08 (0.000 to 0.15)	NA	−0.02 (−0.09 to 0.06)	0.04 (−0.0004 to 0.08)	−0.0002 (−0.08 to 0.08)	0.02 (−0.04 to 0.09)
Evacuation experience before Hurricane Irma	0.07 (−0.08 to 0.22)	NA	−0.01 (−0.10 to 0.09)	0.04 (−0.04 to 0.12)	−0.07 (−0.17 to 0.04)	0.03 (−0.07 to 0.13)
Hours of Hurricane Irma–related media exposure	0.01 (0.01 to 0.02)^d^	NA	0.005 (−0.003 to 0.01)	0.01 (0.003 to 0.01)^d^	0.003 (−0.003 to 0.01)	0.01 (0.004 to 0.02)^d^
Female^e^	0.09 (−0.01 to 0.18)	NA	0.01 (−0.07 to 0.09)	0.05 (−0.002 to 0.09)	0.05 (−0.04 to 0.13)	0.05 (−0.03 to 0.13)
College education	0.003 (−0.07 to 0.08)	NA	−0.03 (−0.10 to 0.04)	0.002 (−0.04 to 0.04)	−0.02 (−0.09 to 0.06)	−0.02 (−0.09 to 0.04)
Race and ethnicity^f^						
Black, non-Hispanic	−0.03 (−0.17 to 0.12)	NA	0.13 (−0.01 to 0.27)	−0.01 (−0.09 to 0.06)	−0.09 (−0.27 to 0.09)	0.10 (−0.03 to 0.23)
Hispanic	−0.003 (−0.12 to 0.11)	NA	0.02 (−0.08 to 0.12)	−0.002 (−0.06 to 0.06)	−0.06 (−0.15 to 0.04)	0.02 (−0.07 to 0.10)
Other, non-Hispanic^g^	0.19 (−0.10 to 0.47)	NA	−0.14 (−0.27 to −0.01)	0.10 (−0.05 to 0.25)	−0.05 (−0.24 to 0.15)	−0.03 (−0.19 to 0.13)
Income	−0.03 (−0.04 to −0.02)^c^	NA	−0.01 (−0.02 to 0.004)	−0.02 (−0.02 to −0.01)^c^	−0.005 (−0.02 to 0.01)	−0.02 (−0.03 to −0.01)^c^
**Wave 2**						
In evacuation zone during Hurricane Irma and did not evacuate^h^	0.16 (0.03 to 0.29)^i^	NA	0.02 (−0.07 to 0.12)	0.09 (0.02 to 0.15)^i^	0.14 (0.03 to 0.24)^i^	0.10 (0.003 to 0.19)^i^
In evacuation zone during Hurricane Irma and evacuated^h^	0.08 (−0.05 to 0.20)	NA	0.07 (−0.02 to 0.15)	0.04 (−0.03 to 0.11)	0.04 (−0.07 to 0.14)	0.10 (0.01 to 0.19)^i^
Loss and/or injury in Hurricane Irma	0.16 (0.06 to 0.26)^d^	NA	0.02 (−0.07 to 0.10)	0.09 (0.03 to 0.14)^d^	−0.001 (−0.09 to 0.09)	0.09 (0.003 to 0.17)^i^
Global distress	NA	NA	0.54 (0.44 to 0.63)^c^	NA	0.01 (−0.11 to 0.12)	0.46 (0.36 to 0.56)^c^
**Wave 3**						
Direct exposure to Hurricane Michael	NA	NA	0.09 (−0.09 to 0.27)	NA	0.10 (−0.10 to 0.31)	0.08 (−0.08 to 0.24)
Indirect exposure to Hurricane Michael	NA	NA	0.05 (−0.002 to 0.10)	NA	0.07 (0.02 to 0.12)^d^	0.04 (−0.003 to 0.09)
Hours of Hurricane Michael–related media exposure	NA	NA	0.001 (−0.01 to 0.01)	NA	0.02 (0.01 to 0.02)^c^	0.001 (−0.01 to 0.01)
Recent individual-level adversity	NA	NA	0.03 (0.004 to 0.05)^i^	NA	−0.01 (−0.03 to 0.02)	0.02 (0.003 to 0.04)^i^
Global distress	NA	NA	NA	NA	0.86 (0.74 to 0.99)^c^	NA

Abbreviation: NA, not applicable.

^a^ Data are for 1637 Florida residents in the total sample. Florida residents were surveyed in the 60 hours before Hurricane Irma (wave 1: September 8-11, 2017). A second survey was administered 1 month after Hurricane Irma (wave 2: October 12-29, 2017), and a third survey was administered after Hurricane Michael (wave 3: October 22 to November 6, 2018).

^b^ A response of 0 indicated no prior mental health ailments; 1, prior anxiety or depression; and 2, prior anxiety and depression.

^c^ *P* < .001.

^d^ *P* < .01.

^e^ Male was the reference group.

^f^ White, non-Hispanic was the reference group.

^g^ Other included those who identified as American Indian or Alaska Native, Asian, Native Hawaiian or other Pacific Islander, or “a different race.”

^h^ Not in an evacuation zone was the reference group.

^i^ *P* < .05.

## Discussion

To our knowledge, this is the first study to assess individuals immediately before a category 5 hurricane and follow them longitudinally to assess responses in the immediate aftermath of 2 successive hurricanes (Irma and Michael). This allowed exposure and response assessment with lower than typical bias,^47^ including real-time media exposure to Hurricane Irma. Lowering assessment biases is imperative given recent findings that survey research with nonrepresentative samples has critical biases including inflated point estimates^48^; alternatively, research with representative samples provides more accurate estimates of exposure and psychological responses. We addressed key limitations identified in a recent review of the public health implications of exposure to multiple disasters, most notably the association between repeated exposure to multiple disasters and psychological and physical health.^49^

Unlike previous studies of natural hazards suggesting habituation effects,^50^ this study’s results demonstrated cumulative effects after repeated hurricane exposures. Rather than acclimation to disasters over time, the findings showed associations between direct, indirect, and media-based hazard exposures and increases in mental health problems and functional impairment in work and social settings. This suggests that sensitization processes occurred over time. Hurricane Irma–related PTSS were associated with greater Hurricane Michael–related PTSS and functional impairment. These results align with previous research conducted after repeated exposure to terrorist attacks^22^ and earthquakes^51^ that showed additive effects of repeated disaster exposure associated with mental health symptoms. These findings have critical policy implications; clinicians and policy makers should prepare for the deleterious mental and physical health outcomes that may occur as climate-related hazards increase in frequency and severity.

Key hurricane-related stressors were associated with postevent responses and varied across outcomes, like prior work.^52^ Hurricane-related property loss and/or injury both before and during Hurricane Irma and direct exposure to Hurricane Michael were positively associated with distress responses, similar to Hurricane Katrina–related findings.^13^ Our results support prior research showing associations between these stressors and short-term outcomes,^9^ demonstrating that these associations persisted over time and may sensitize individuals to respond more negatively to subsequent hurricanes. This occurred in the sample in our study even though many respondents were not in the direct path of Hurricane Michael, demonstrating the importance of indirect (ie, knowing someone affected) and media-based exposure. Prevalence rates were similar to those indicated in prior studies of hurricane survivors,^8^ although many respondents in the sample in our study were indirectly exposed. Media exposure to hurricanes Irma and Michael was associated with immediate posthurricane distress; the findings again suggest additive effects. The association between media exposure and distress was likely cyclical; prior work^53^ demonstrated that high levels of media exposure to collective trauma correlated with distress, which in turn correlated with more media exposure and more distress following subsequent events. Taken together, this study’s findings highlight the importance of broad-based approaches to postdisaster outreach because people who experience indirect exposure,^54^ less direct exposure, or primarily media-based exposure may also be at risk for psychological difficulties.^55^

Prehurricane mental health problems and other non–hurricane-related stressors were also associated with increased hurricane-related distress over time. This is particularly important to address in postdisaster outreach. While people with preexisting mental health problems may experience greater postevent mental health symptoms, they are also likely to have their treatment interrupted and not initiate new treatment after a hurricane.^56^ Creating policies that bridge treatment from before to after a disaster and offer community-based resources for these individuals may help break the cycle of distress, particularly in the context of repeated exposure. Similarly, individuals with concurrent non–hurricane-related stressors also reported more distress. Making resources available that address contextual factors (eg, abuse, illness) may also facilitate posthurricane recovery.

### Limitations

This study has limitations. Although the sample was drawn from residents across Florida and population weights were used to increase representativeness, those who were most severely affected by Hurricane Michael comprised a relatively small proportion of the sample. While we were able to maintain a high rate of retention in the follow-up waves, there may have been differences between wave 1 respondents and initial nonresponders, including severity of previous hurricane exposure. Nevertheless, our overall response rate for wave 1 was comparable with those typically seen in disaster studies^9^ and high when accounting for the small sampling time frame (60 hours prior to Hurricane Irma’s landfall).

## Conclusions

In this survey study, repeated direct, indirect, and media-based exposures to hurricanes were associated with increased mental health symptoms over time among Florida residents who experienced hurricanes Irma and Michael. The findings suggest that survey designs that include preassessments and are fielded quickly after a disaster can yield critical insights into longitudinal postevent responses. Recovery from natural hazards may be a protracted process; psychological distress may persist and be exacerbated by subsequent exposures. This study’s results highlight the need to address the mental health implications of repeated exposure to natural hazards, particularly in areas such as the Gulf Coast that are at high risk for repeated hurricane exposure, as risk of hurricanes and other climate-related threats is expected to increase.

## Notes

Supplement. **eTable 1.** Demographic Characteristics of the Sample Compared With Population Benchmarks **eTable 2.** Descriptive Statistics for Posthurricane Posttraumatic Stress, Generalized Worries, Global Distress, and Functional Impairment **eFigure 1.** Map of Survey Respondents Across Florida (Shifted Latitude and Longitude) **eFigure 2.** Direct and Indirect Associations Between Prestorm Factors, Storm Exposures, Posthurricane Generalized Worries, and Functional Impairment **eFigure 3.** Direct and Indirect Associations Between Prestorm Factors, Storm Exposures, Posthurricane Global Distress, and Functional Impairment
