# Supplementary material for: Association Between Repeated Exposure to Hurricanes and Mental Health in a Representative Sample of Florida Residents
Source: JAMA Netw Open. 2022 Jun 16;5(6):e2217251. doi: 10.1001/jamanetworkopen.2022.17251 (PMC9204543; doi:10.1001/jamanetworkopen.2022.17251)
Supplement: Supplement. — eTable 1. Demographic Characteristics of the Sample Compared With Population Benchmarks eTable 2. Descriptive Statistics for Posthurricane Posttraumatic Stress, Generalized Worries, Global Distress, and Functional Impairment eFigure 1. Map of Survey Respondents Across Florida (Shifted Latitude and Longitude) eFigure 2. Direct and Indirect Associations Between Prestorm Factors, Storm Exposures, Posthurricane Generalized Worries, and Functional Impairment eFigure 3. Direct and Indirect Associations Between Prestorm Factors, Storm Exposures, Posthurricane Global Distress, and Functional Impairment [file jamanetwopen-e2217251-s001.pdf]

Supplementary Online Content

Garfin DR, Thompson RR, Holman EA, Wong-Parodi G, Silver RC. Association between repeated exposure to hurricanes and mental health in a representative sample of Florida residents. *JAMA Netw Open*. 2022;5(6):e2217251. doi:10.1001/jamanetworkopen.2022.17251

eTable 1. Demographic Characteristics of the Sample Compared with Population Benchmarks

eTable 2. Descriptive Statistics for Posthurricane Posttraumatic Stress, Generalized Worries, Global Distress, and Functional Impairment

eFigure 1. Map of Survey Respondents Across Florida (Shifted Latitude and Longitude)

eFigure 2. Direct and Indirect Associations Between Prestorm Factors, Storm Exposures, Posthurricane Ongoing Worries, and Functional Impairment

eFigure 3. Direct and Indirect Associations Between Prestorm Factors, Storm Exposures, Posthurricane Global Distress, and Functional Impairment

This supplementary material has been provided by the authors to give readers additional information about their work.

© 2022 Garfin DR et al. *JAMA Network Open.*

eTable 1. *Demographic Characteristics of the Sample Compared with Population Benchmarks*

	**KnowledgePanel Florida Sample (*N*=1,637)**		**Adult FL Population American Community Survey 2015 (*N*=15,752,541)**
**Demographic Variables**	**Weighted *n***	**Weighted %**	**%**
Gender by Age			
Male 18-29	84	5.15	9.40
Female 18-29	145	8.88	9.28
Male 30-44	187	11.42	11.25
Female 30-44	205	12.55	11.83
Male 45-59	215	13.11	12.32
Female 45-59	230	14.07	13.28
Male 60 +	258	15.74	14.79
Female 60 +	313	19.09	17.86
Race/Ethnicity[a]			
White, non-Hispanic	1,007	61.53	58.40
Black (African American), non-Hispanic	201	12.30	13.93
Other, non-Hispanic	55	3.34	3.14
Hispanic	352	21.50	23.29
2 + races, non-Hispanic	22	1.33	1.23
Metro			
Metro	1582	96.66	96.21
Nonmetro	55	3.34	3.79
Level of Education			
Less than/high school graduate	619	37.83	41.42
Some college	547	33.43	31.65
Bachelor’s degree or above	471	28.74	26.93
Income			
Under $25,000	295	18.01	17.89
$25,000-$49,999	418	25.54	24.65
$50,000-$74,999	340	20.78	20.08
$75,000-$99,999	226	13.78	13.28
$100,000-$149,999	232	14.20	13.61
$150,000 and over	126	7.70	10.48

[a]“Other” includes those who identify as American Indian or Alaska Native, Asian, Native Hawaiian or other Pacific Islander, or “a different race.”

© 2022 Garfin DR et al. *JAMA Network Open.*

eTable 2. *Descriptive Statistics for Post-Hurricane Posttraumatic Stress, Generalized Worries, Global Distress, and Functional Impairment, N=1,637*

**Variables**	Wave 2 (*M* [SD]; Range)	Wave 3 (M[SD]; Range)
Posttraumatic stress symptoms	1.48 [0.71]; 1-5	1.48 [0.69]; 1-4.8
Generalized worries	2.09 [0.86]; 1-5	2.04 [0.81]; 1-5
Global distress	0.49 [0.66]; 0-4	0.47 [0.61]; 0-4
Functional impairment	--	1.54 [0.80]; 1-5]
Probable PTSD[a]	10.97% (*n*=166)	10.92% (*n*=121)

Note: Posttraumatic stress symptoms measured using the Primary Care PTSD Screen for the DSM-5.[32] Generalized worries was measured using 8 items assessing past-week worries about the possibility of a) a terrorist attack, b) a natural disaster, c) violence (shootings, stabbings, or physical assault), and d) financial stress or strain.[38] Global distress was measured using a modified 9-item version of the Brief Symptom Inventory-18.[36] Functional impairment was measured using 4 items from the SF-36.[38]

[a]Since not all participants met Criterion A for trauma exposure, they are not assumed to have PTSD as indicated by the Diagnostic and Statistical Manual of Mental Disorders, 5th edition.

© 2022 Garfin DR et al. *JAMA Network Open.*

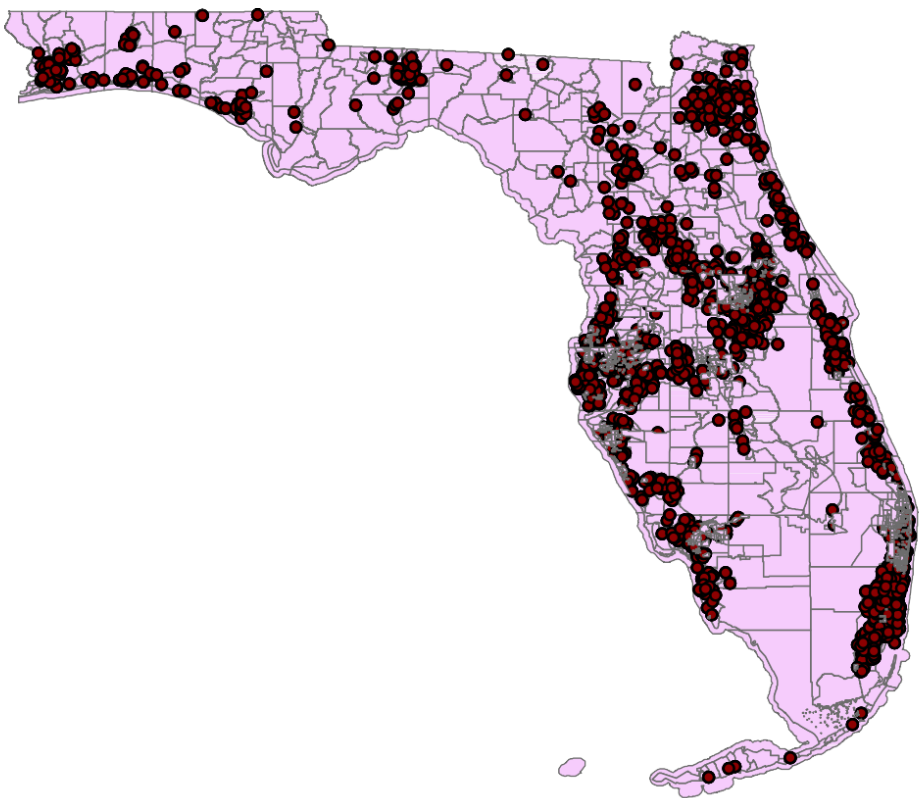

eFigure 1. Map of Survey Respondents Across Florida (Shifted Latitude and Longitude)

© 2022 Garfin DR et al. *JAMA Network Open.*

eFigure 2. Direct and Indirect Associations Between Prestorm Factors, Storm Exposures, Posthurricane Generalized Worries, and Functional Impairment

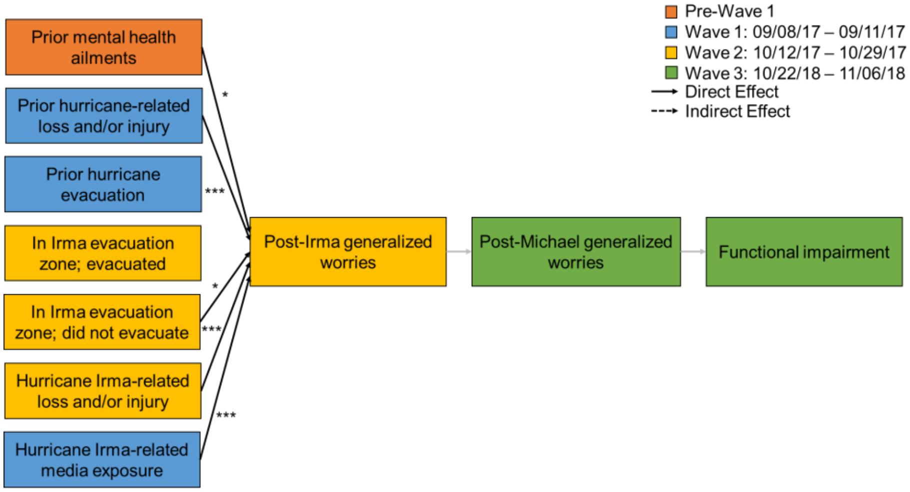

eFigure 2a. Predictors of wave 2 generalized worries

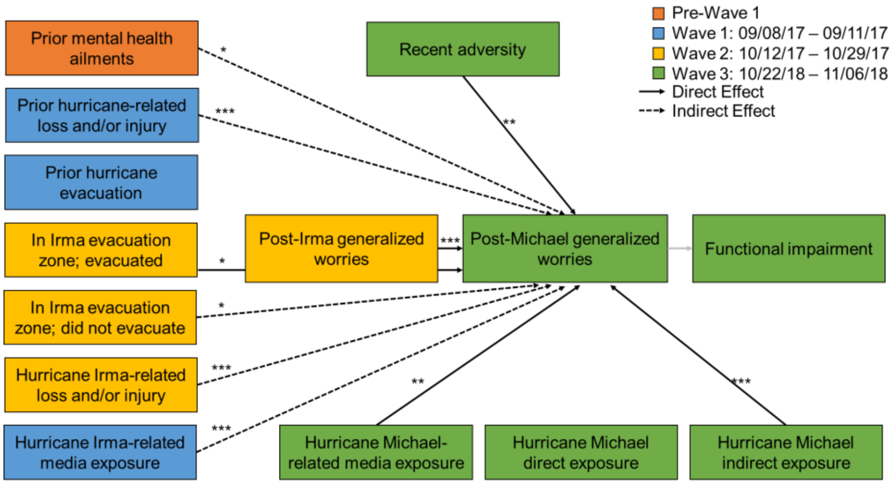

eFigure 2b. Predictors of wave 3 generalized worries

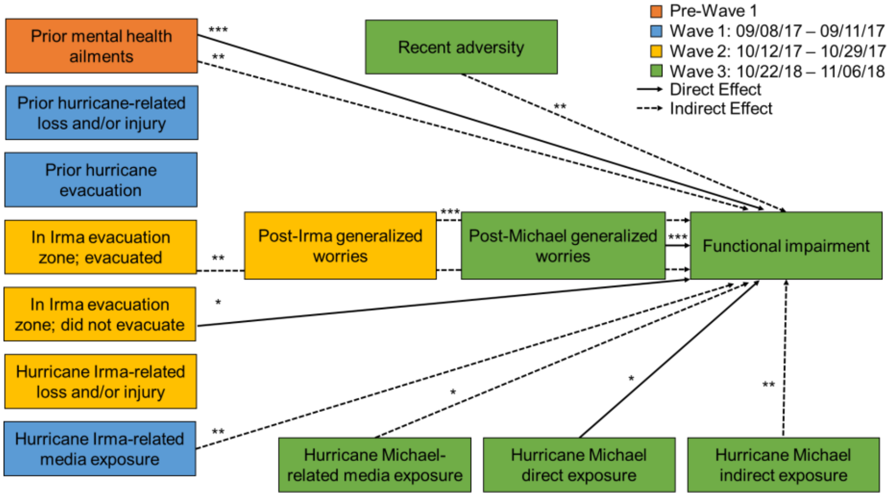

eFigure 2c. Predictors of wave 3 functional impairment

Note: * *p* < .05; ** *p* < .01; *** *p* < .001

© 2022 Garfin DR et al. *JAMA Network Open.*

eFigure 3. Direct and Indirect Associations Between Prestorm Factors, Storm Exposures, Posthurricane Global Distress, and Functional Impairment

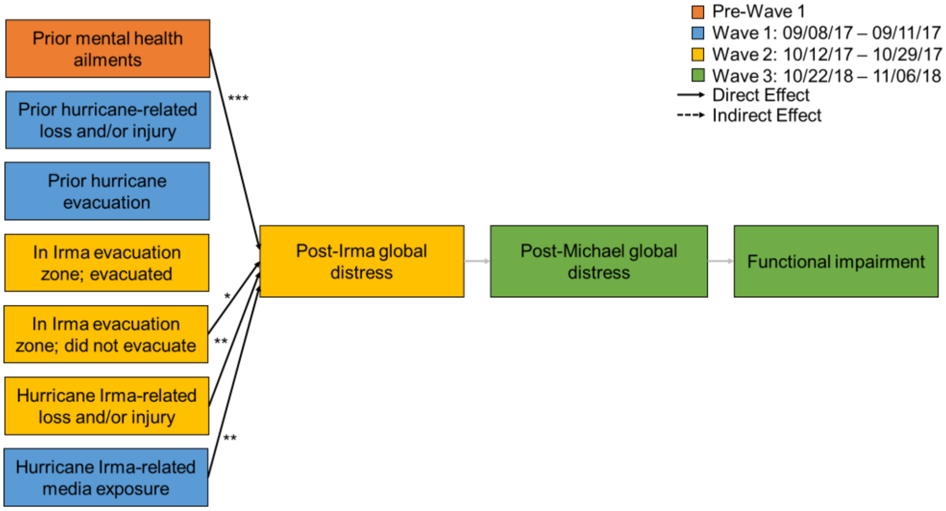

eFigure 3a. Predictors of wave 2 global distress

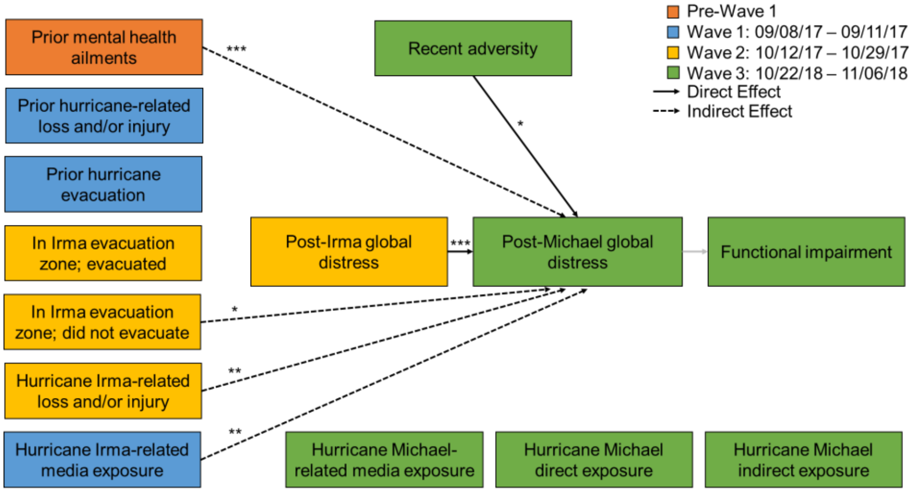

eFigure 3b. Predictors of wave 3 global distress

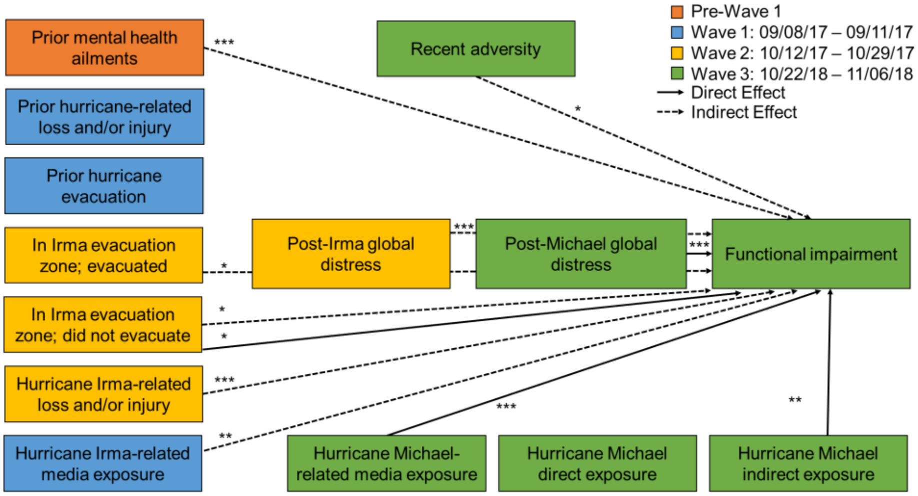

eFigure 3c. Predictors of wave 3 functional impairment

Note: * $p < .05$; ** $p < .01$; *** $p < .001$

© 2022 Garfin DR et al. *JAMA Network Open.*
